# MEMS Resonant Cantilevers for High-Performance Thermogravimetric Analysis of Chemical Decomposition

**DOI:** 10.3390/s23136147

**Published:** 2023-07-04

**Authors:** Zhi Cao, Hao Jia, Yufan Zhou, Ming Li, Pengcheng Xu, Xinxin Li, Dan Zheng

**Affiliations:** 1 School of Chemical and Environmental Engineering, Shanghai Institute of Technology, Shanghai 201418, China; 216062101@mail.sit.edu.cn; 2 State Key Lab of Transducer Technology, Shanghai Institute of Microsystem and Information Technology, Chinese Academy of Sciences, Shanghai 200050, China; zhouyf@mail.sim.ac.cn (Y.Z.); liming01@mail.sim.ac.cn (M.L.); xpc@mail.sim.ac.cn (P.X.); xxli@mail.sim.ac.cn (X.L.); 3 School of Microelectronics, University of Chinese Academy of Sciences, Beijing 100049, China

**Keywords:** MEMS, resonant cantilever, TGA, heating rate, thermal lag

## Abstract

We investigate the MEMS resonant cantilevers for high-performance thermogravimetric analysis (TGA) of chemical decomposition, featuring high accuracy and minimized thermal lag. Each resonant cantilever is integrated with a microheater for sample heating near the free end, which is thermally isolated from the resonance excitation and readout elements at the fixed end. Combining finite element modeling and experiments, we demonstrate that the sample loading region can stabilize within ~11.2 milliseconds in response to a step heating of 500 °C, suggesting a very fast thermal response of the MEMS resonant cantilevers of more than 10^4^ °C/s. Benefiting from such a fast thermal response, we perform high-performance TG measurements on basic copper carbonate (Cu_2_(OH)_2_CO_3_) and calcium oxalate monohydrate (CaC_2_O_4_·H_2_O). The measured weight losses better agree with the theoretical values with 5–10 times smaller thermal lags at the same heating rate, compared with those measured by using conventional TGA. The MEMS resonant cantilevers hold promise for highly accurate and efficient TG characterization of materials in various fields.

## 1. Introduction

Thermogravimetric analysis (TGA) has been a prevailing technique for the thermal characterization of a variety of materials, such as composites, polymers, metals, and drugs. When an analyte is subject to a programmed temperature increase or decrease (in the ambient or specific gas), the analyte may thermally decompose or react with the gas, leading to mass changes at specific temperature values. Therefore, the TGA curves can reveal important information about thermal stability and chemical reaction processes at characteristic temperatures of different materials [1,2]. Given the thermal decomposition as an example, conventional TG apparatus measures the weight loss by utilizing a high-precision balance surrounded by a heated furnace. The mass resolution (*δm*) of the balance is down to ~0.1 micrograms (μg), and 1–10 milligram (mg) sample amounts are often required. More importantly, an appropriate heating rate is critical to achieve uniform sample heating inside the furnace and to minimize thermal lags. Different heating rates result in temperature gradients between the interior and exterior of the sample [3]. Even for commonly used heating rates, such as 1–40 °C/min, significant thermal lags often occur in practice, e.g., the measured peak temperature could increase by ~20–75 °C as the heating rate increases [4,5,6,7]. Therefore, achieving rapid and uniform heating to ensure high-accuracy yet high-efficient TG measurements is still challenging [8,9].

To address the limitations in the furnace-based conventional TGA, chip-scale TGA utilizing temperature-controlled micro-electromechanical systems (MEMS) resonant cantilevers have been proposed [10,11,12,13,14]. Quite different from the conventional TGA that measures weight loss by using a balance during heating, the resonant cantilevers directly monitor the mass change (Δ*m*) by using resonance frequency shift (Δ*f*), based on the relationship that Δ*f* is linearly proportional to Δ*m* [15]. However, realizing MEMS TGA towards real-world applications has been quite challenging when taking into account the design and fabrication of fully integrated devices (with on-chip resonance excitation/readout, heating/temperature readout elements), and the improvement in heating performance. For example, commercial SPM probes were first introduced for TGA measurement, however, this type of device was excited using an off-chip piezo shaker. The resonance readout and heating shared the same piezoresistor, which limited the temperature range to ~530 °C [10]. Heater-integrated cantilevers were also proposed, while both the resonance excitation and readout were achieved by using a bulky and expensive laser system [11]. Although later, a paddle-shaped silicon nitride cantilever integrated with a polysilicon heater and thermocouple was introduced, such devices still showed a limited temperature range of up to 650 °C [12]. More recently, our group has developed fully integrated MEMS resonant cantilevers with a much higher temperature range of >1000 °C [13,14]. Thanks to the miniature device size, the resonant cantilevers also exhibit an ultrahigh mass resolution down to sub-picogram (10^−12^ g), and only nanogram (10^−9^ g) samples are required [16,17].

Despite the aforementioned achievements in the design and fabrication of fully integrated devices for TGA, another important aspect, i.e., the thermal response and thermal lag effect of the cantilever-TGA under various heating rates have been rarely modeled and discussed. Therefore, in this work, combing transient finite element modeling and experiments, we take the initiative to quantitatively analyze our MEMS cantilever-based TGA with good accuracy and minimized thermal lags.

## 2. Modeling of Thermal Response of MEMS Resonant Cantilevers

**Figure 1:**
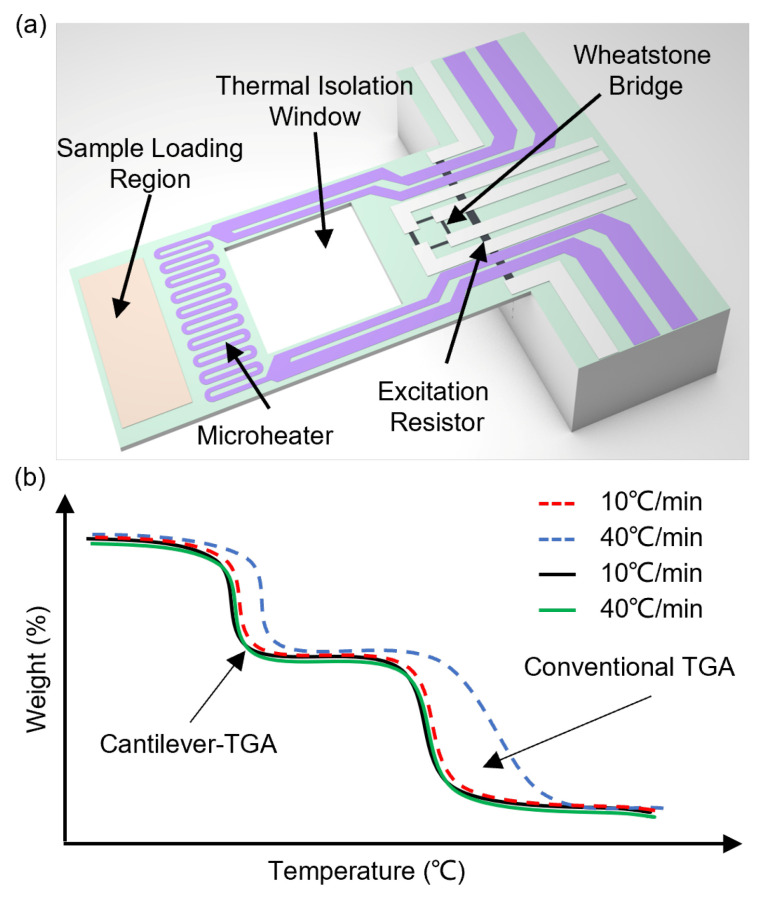
Illustration of MEMS resonant cantilever-based TGA. (**a**) Schematic illustration of the cantilever-TGA chip. The mass change of the analyte is measured by the resonance frequency change of the cantilever. (**b**) Compared with conventional TGA, cantilever-TGA features minimized thermal lag at the same heating rate.

Figure 1 illustrates the temperature-controlled MEMS resonant cantilever [14]. The resonant cantilever has on-chip integrated resonance excitation and readout resistors at the cantilever fixed end and a microheater for sample heating near the free end. When the sample loading region is heated up to hundred degrees centigrade by the microheater, the heat also tends to transfer through the solid to the fixed end, which may affect the normal operation of excitation and readout resistors that are made of highly doped silicon. Therefore, a thermal isolation window is specially designed in the middle of the cantilever. The cantilever resonance is electrothermally excited and piezoresistively read out by the Wheatstone bridge [14,18,19]. Each of the 4 resistors has a resistance of ~800 Ω. The input voltage for the Wheatstone bridge is 2.6 V, which is kept the same during the heating process. The piezoresistive elements could be influenced by the temperature, but a Wheatstone bridge composed of four resistors with identical resistance values can theoretically suppress the impact of temperature variations [20]. We also design the thermal isolation window to avoid overheating the Wheatstone bridge, to minimize the temperature-induced frequency shift. Meanwhile, although there is a temperature-induced frequency shift due to the heating of the Wheatstone bridge, the TG curves are obtained with baseline correction, in which this effect could be minimized. 

The principle of resonant mass sensing can be detailed as follows. Theoretically, the resonance frequency (*f*_0_) of a resonator is given by $f_{0}=1/2\pi \sqrt{k/m}$, where *k* and *m* are the effective spring constant and effective mass of the resonator. For a cantilever, *k* = *Ebh*^3^/4*l*^3^, where *l*, *b*, and *h* are the length, width, and thickness, respectively. *E* is Young’s modulus. *m* ≈ 0.236*ρlbh*, where *ρ* is the density. When a trace amount of analyte (Δ*m* << *m*) is loaded onto the resonator, the resonance frequency will shift (i.e., $f_{1}=1/2\pi \sqrt{k/\left(m+\Delta m\right)}$) in response to the added mass in a linear relationship, where ℜ *=* $\Delta f/\Delta m=\left(-f_{0}/2m\right)$ is the mass responsivity of the resonator [21,22,23]. ℜ is calibrated to be ~0.24 Hz/pg for the resonant cantilevers in this work by using standard PS microspheres.

To obtain a thermogravimetric curve, we first measure the resonance frequency as a function of the temperature (*f*_0_ vs. *T*) for an empty cantilever without a sample. The resonance frequency of the empty cantilever at room temperature is *f*_00_, and the resonance frequency of the empty cantilever when the sample loading region is heated to a given temperature *T* is *f*_0T_. Subsequently, we load a few nanograms of the sample (Δ*m_0_*, typically a few nanograms) to the cantilever by using a customized microinjection system. When the sample loading region is heated from room temperature to a given high temperature *T*, we record the resonance frequency as a function of the temperature (*f*_1_ vs. *T*) for the loaded cantilever. The resonance frequency of the loaded cantilever at room temperature is *f*_10_, and the resonance frequency of the loaded cantilever when the sample loading region is heated to a given temperature *T* is *f*_1T_. Therefore, the ratio between the remaining mass at *T* (Δ*m_T_*) and the loaded sample mass (Δ*m*_0_) can be plotted as a TG curve (Equation (1)), based on a reasonable assumption that the temperature effect on the spring constant of the cantilever is negligible [24].

(1) $$\frac{\Delta m_{T}}{\Delta m_{0}}=\frac{1/f_{1T}{}^{2}-1/f_{0T}{}^{2}}{1/f_{10}{}^{2}-1/f_{00}{}^{2}}$$

**Figure 2:**
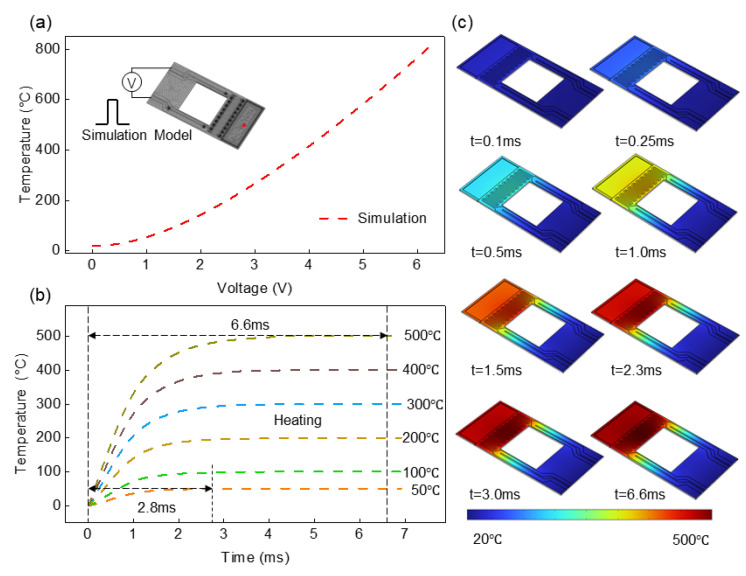
Temperature response of the MEMS resonant cantilevers under step heating voltage. (**a**) Simulated temperature in the center of the sample loading region vs. heating voltage. (**b**) Transient thermal response of the cantilever under different heating steps of 50–500 °C. (**c**) Transient response of the cantilever under heating steps of 500 °C.

To determine whether the cantilever-TGA is advantageous over conventional TGA in high heating rates with minimized thermal lags, we investigate the transient temperature response of the MEMS resonant cantilevers using finite element modeling. In the COMSOL model (Figure 2a), the cantilever is designed to have a length of 290 μm, and a width of 140 μm, with the thermal isolation window to be 100 × 100 μm. The cantilever has a 3-μm-thick silicon (Si) supporting layer defined by the SOI wafer. A thin dielectric layer for passivating the electrodes is omitted to simplify the model since it does not play a critical role in the thermal response of the cantilever. The model consists of two modules: an electric current module of the molybdenum (Mo) microheater to create joule heating by adding a heating voltage (*V*_h_), and a heat transfer module to resolve temperature distribution in the sample loading region. The thermal properties of the Si and Mo used in the model are summarized in Table 1. We apply a step heating voltage to the microheater to create a temperature increase in the sample loading region. By using a time-dependent solver, we simulate and analyze the dynamic response of the cantilever-TGA chip.

Figure 2b shows the simulated relationship between the heating voltage and temperature in the center of the sample loading region (*V*_h_ vs. *T*). According to the transient analysis, we observe that a heating voltage of *V*_h_ ~4.6 V can lead to a sharp temperature increase of ~500 °C in the sample loading region, and it only takes ~6.6 milliseconds for the sample loading region to stabilize. The simulation results suggest efficient and fast heat transfer in the cantilever-TGA chip, due to the good thermal properties of silicon, miniature device size, and thermal isolation design. Meanwhile, based on the simulation results, we theoretically predict the very fast thermal response of the resonant cantilevers of up to ~7.6 × 10^4^ °C/s. It is worth noting that although the response time of the heating voltage source, the control circuitry, and the presence of the analyte is not considered in the model, we would still reasonably expect that the thermal response of the cantilever-TGA is much higher than that of the furnace, given the very small amount of samples of a few nanograms. In other words, given the same heating rate, greatly minimized thermal lags are expected for the cantilever-TGA, compared with the conventional TGA, which will be verified in the experimental section.

## 3. Fabrication of the MEMS Resonant Cantilevers

The MEMS resonant cantilevers are batch-fabricated based on a (100) SOI wafer, which has a 3 μm top silicon layer, 700 nm buried oxygen (BOX) layer, and 380 μm silicon substrate. Ion implantation is used to create the resistors for resonance excitation and detection at the cantilever fixed end. The molybdenum (Mo) heater is patterned using the lift-off method. After the opening of contact holes, 300-nm gold (Au) film is sputtered to form contacts. The wafer surface is then deposited by a 150-nm dielectric layer via plasma-enhanced chemical vapor deposition (PECVD). After removing the dielectric layer using reactive-ion etching (RIE), the cantilever with a thickness of 3 μm is patterned by deep reactive-ion etching (DRIE). The silicon substrate of the SOI wafer is etched from the backside using DRIE. Finally, the BOX layer is removed by buffered oxide etching (BOE) and the cantilevers are suspended.

## 4. Characterization of Thermal Response of MEMS Resonant Cantilevers

**Figure 3:**
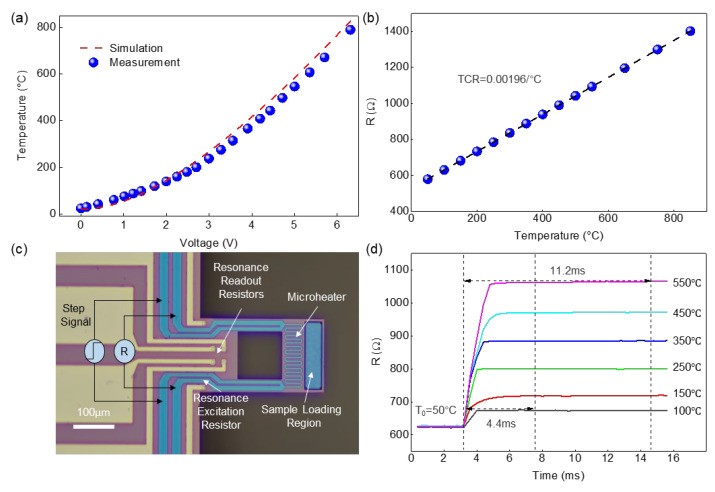
Thermal response of the MEMS resonant cantilevers. (**a**) Measured heating voltage vs. temperature in the center of the sample loading region. The results (scatter points) show reasonable agreement with the simulated results in Figure 2a (dashed line). (**b**) Measured TCR of the Mo heating resistor. The dashed line shows linear fitting of the measured results (scatter points). (**c**) Optical image of the MEMS cantilever and illustration of measuring temperature response. (**d**) Thermal response of the MEMS cantilever under different step heating. It takes ~11.2 ms for the sample loading region to stabilize under step heating from 50 to 550 °C.

The fabricated MEMS cantilever is shown in Figure 3. The resonance excitation and readout resistors are located near the cantilever fixed end and the microheater is near the free end, next to the sample-loading region. To experimentally verify the fast thermal response of the cantilever-TGA chip, we first calibrate the temperature coefficient of resistance (TCR) of the Mo microheater and correlate it with the temperature in the sample loading region. The actual temperature is determined by the infrared camera [25,26]. Figure 3a shows the calibrated relationship between the heating voltage and the temperature in the center of the sample-loading region (*V*_h_ vs. *T*). The measured results (scatter points) show reasonable agreement with the simulated ones (dashed line). The small discrepancy at high temperature up to 800 °C may be attributed to the simplified cantilever structure and temperature-dependent thermal properties in the model. We also calibrate the linear TCR of the Mo microheater to be ~0.00196/°C, as shown in Figure 3b. This TCR value is important for accurately measuring and controlling the temperature during the following cantilever-TGA experiments.

We then measure the transient thermal response of the MEMS cantilever under step heating. According to the simulation results, the cantilever response time for a step heating is several milliseconds; it is challenging to capture such a fast response by using normal thermal imaging or spectroscopy approaches. Since the TCR correlates the temperature of the sample loading region with the Mo resistance, we perform a four-point probe measurement by applying a step heating signal to the Mo resistor while monitoring its resistance change as a function of time using high-speed circuitry, as illustrated in Figure 3c. The temperature of the sample-loading region is first stabilized at ~50 °C. We then set a heating step (e.g., from 50 °C to 550 °C, a heating step of 500 °C), and measure the time it takes for the sample loading region to stabilize at the new temperature value (e.g., 550 °C). Hence, we can experimentally characterize the heating rate of the MEMS cantilever. As shown in Figure 3d, given a heating step from 50 °C to 550 °C, it takes ~11.2 ms for the Mo resistance to change from ~624 Ω to ~1064 Ω and stabilize, suggesting that the sample loading region finally stabilizes at 550 °C. We can thus verify the very fast thermal response of the resonant cantilevers of up to ~4.5 × 10^4^ °C/s. It is worth noting that the measured response time is longer than the simulated value, because in the actual measurements, the response time of the source signal and measurement circuitry are also added to the total response time. Still, we would expect that the heating rate of the cantilever is still much higher than that of the furnace, hence the thermal lag will be greatly minimized in the cantilever-TGA.

We also monitor the cantilever resonance and characterize the temperature-dependent shift in resonance frequency. For a typical MEMS resonant cantilever fabricated in this work, the shift of resonance frequency during heating from room temperature to 500 °C is within 100 Hz, corresponding to a temperature coefficient of frequency, Tc*f* ~0.2 Hz/°C (3.9 ppm/°C). Such value is much smaller compared with those from MEMS cantilever-based TGA in the literature [11,12]. On the other hand, although there is a temperature-induced frequency shift during heating, the TG curves are obtained according to Equation (1), in which this effect can be minimized.

Another advantage of the MEMS cantilever-based TGA is the low power consumption. We measure the power for heating the cantilever sample loading region to different temperature values, as shown in Table 2. It can be observed that when the cantilever sample loading region is heated up to 500 °C, the heating power is only ~21.4 mW. This value is orders of magnitude lower than that of the conventional TGA, which is typically up to hundreds of watts.

## 5. TG Measurements Using MEMS Resonant Cantilevers

**Figure 4:**
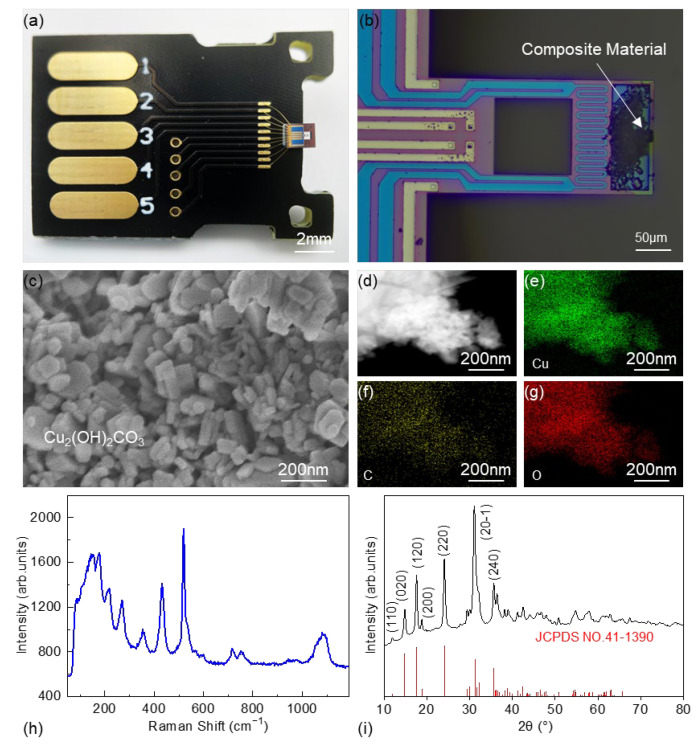
Cantilever-TGA chip with loaded Cu_2_(OH)_2_CO_3_. (**a**,**b**) Optical image of the cantilever-TGA chip after wire bonding and sample loading. (**c**) The SEM image showing the morphology of Cu_2_(OH)_2_CO_3_. (**d**–**i**) EDS mapping, Raman spectrum, and XRD plot confirming the high-quality Cu_2_(OH)_2_CO_3_ crystals.

Figure 4a shows a typical cantilever-TGA chip for sample loading. The chip is packaged on a PCB substrate and wire-bonded for testing. The PCB substrate has 10 gold electrodes on both sides, enabling direct insertion into a 10-pin board-to-board connector for easy plug-and-play. As illustrated in Figure 4b, the lateral dimensions of the cantilever are 290 μm × 140 μm, and the composite materials are loaded in the sample loading region. 

As to the TG measurements, taking chemical decomposition as an example, we choose two composite materials, which have been widely used for evaluating TG apparatus. Basic copper carbonate (Cu_2_(OH)_2_CO_3_) is a fine powder with a peacock green color, which decomposes upon heating into a black powder. Calcium oxalate monohydrate (CaC_2_O_4_·H_2_O) is a white, crystalline powder. The powder is dispersed in ethylene glycol. A microinjection system is specifically designed for MEMS device sample loading. This system enables micrometer-level positioning accuracy and nanoliter-level control of injection volume, ensuring accurate and gentle injection to the sample loading region. Subsequently, the chip is placed in an 80 °C oven for half an hour to allow complete evaporation of the ethylene glycol. As shown in Figure 4b, after the solvent has completely evaporated, the sample is distributed within the sample loading region.

We also confirm the high-quality crystals by using scanning electron microscopy (SEM) and transmission electron microscopy (TEM). Taking Cu_2_(OH)_2_CO_3_ as an example, the SEM image in Figure 4c shows that the Cu_2_(OH)_2_CO_3_ particles exhibit predominantly rectangular shapes, with a length ranging from approximately 0.1–0.4 μm. Elemental energy-dispersive spectroscopy (EDS) is conducted and presented in Figure 4d–g, showing distributions of Cu, C, and O elements in the material. Furthermore, Raman spectroscopy is employed to characterize the material, as shown in Figure 4h, and the obtained Raman spectrum exhibits characteristic peaks consistent with those in the RRUFF spectroscopy database (RRUFF ID: R050531) [27]. Additionally, the position and intensity of the X-ray diffraction (XRD) peaks match those of the standard card (JCPDS NO. 41-1390) for Cu_2_(OH)_2_CO_3_. These results collectively confirm the high quality and purity of the samples, such that the measured weight losses via cantilever-TGA and conventional TGA can be directly evaluated with theoretical values derived from the chemical equations.

### 5.1. TG Measurements of Basic Copper Carbonate Decomposition

**Figure 5:**
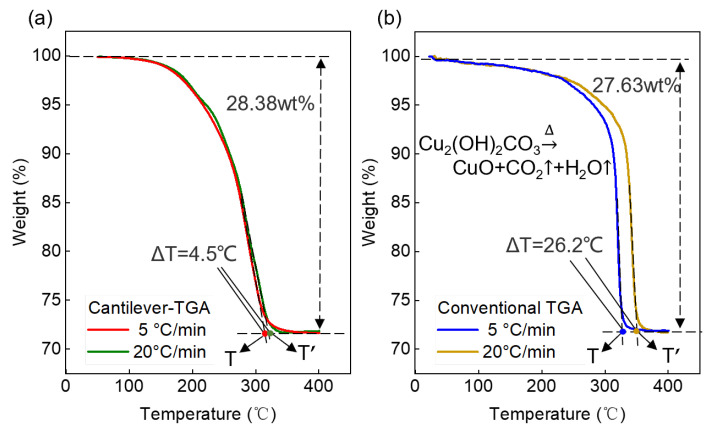
TG measurements of Cu_2_(OH)_2_CO_3_ decomposition at two different heating rates of 5 °C/min, and 20 °C/min using (**a**) cantilever-TGA and (**b**) conventional TGA.

Figure 5 presents the TG curves of Cu_2_(OH)_2_CO_3_ obtained using cantilever-TGA and conventional TGA. Two different heating rates of 5 °C/min and 20 °C/min are chosen to evaluate the thermal lag effect. Theoretically, Cu_2_(OH)_2_CO_3_ gradually decomposes into CuO, CO_2,_ and H_2_O in two steps under programmed heating up to 400 °C, with a total weight loss of ~28.1%, according to the equation. In the measurements using our cantilever-TGA (Figure 5a, also Table 3), we obtain total weight loss values of 28.30% and 28.38% at 5 °C/min and 20 °C/min, respectively, showing deviations of 0.7% and 1.0% from the theoretical value. On the other hand, in the measurements using conventional TGA (Figure 5b, also Table 3), we obtain total weight loss values of 27.74% and 27.63% at 5 °C/min and 20 °C/min, respectively, showing deviations of −1.3% and −1.7% from the theoretical value. Therefore, the weight losses measured by the cantilever-TGA are closer to the theoretical value, suggesting better accuracy than conventional TGA.

Additionally, we evaluate the thermal lag effect based on the termination temperature of the thermal decomposition (Figure 5, also Table 4). In the measurements using cantilever-TGA, we obtain termination temperature values of 318.2 °C and 322.7 °C at 5 °C/min and 20 °C/min, respectively, hence a difference of 4.5 °C. In contrast, in the measurements using conventional TGA, we obtain termination temperature values of 324.4 °C and 350.6 °C at 5 °C/min and 20 °C/min, respectively, showing a more than 6-fold larger difference (26.2 °C). The results indicate that by using cantilever-TGA, the thermal lag effect at higher heating rates can be greatly minimized. Therefore, it can be verified that cantilever-TGA is advantageous over conventional TGA in terms of smaller thermal lag.

### 5.2. TG Measurements of Calcium Oxalate Monohydrate Decomposition

We then perform TG measurements on the CaC_2_O_4_·H_2_O standard sample, which features a three-stage decomposition. This will help to verify the high performance of cantilever-TGA for characterizing more complicated multi-step processes. During programmed heating up to 800 °C, the CaC_2_O_4_·H_2_O first decomposes into calcium oxalate (CaC_2_O_4_) by losing H_2_O. The CaC_2_O_4_ then decomposes into calcium carbonate (CaCO_3_) and releases carbon monoxide (CO), and finally, the CaCO_3_ decomposes into calcium oxide (CaO) and releases carbon dioxide (CO_2_). Therefore, the theoretical weight losses are 12.32%,19.16%, and 30.11%, respectively.

**Figure 6:**
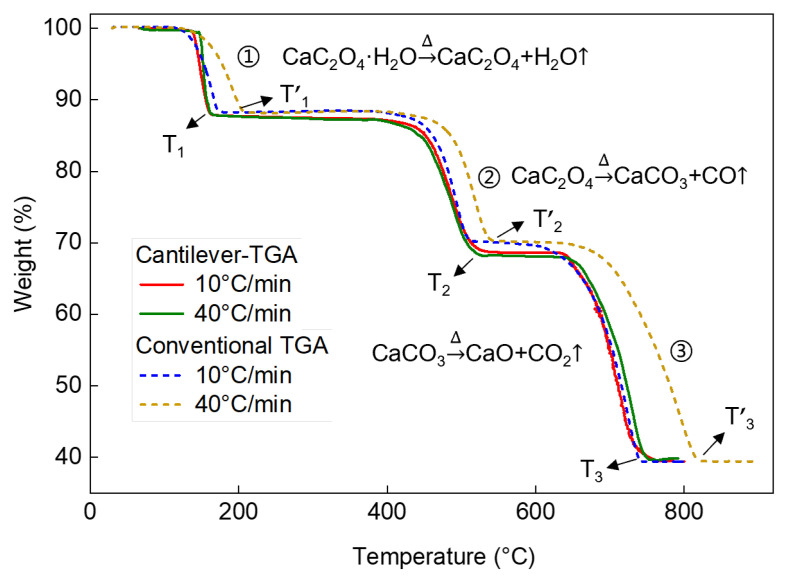
TG measurements of CaC_2_O_4_·H_2_O decomposition at two different heating rates of 10 °C/min, and 40 °C/min using cantilever-TGA vs. conventional TGA.

Using our cantilever-TGA, we observe three distinct decomposition stages during programmed heating to 800 °C (Figure 6, also Table 5). The weight losses corresponding to the three-step decomposition are measured to be 12.18%, 19.57%, 28.63% at 40 °C/min. The deviations from theoretical values are −1.1%, 2.1%, and −4.9%, respectively. In contrast, for conventional TGA, the measured weight losses are 11.34%, 18.21%, and 31.10% at 40 °C/min. The deviations from theoretical values are −8.0%, −5.0%, and 3.3%, larger than those measured by the cantilever-TGA. Again, the results suggest better accuracy of cantilever-TGA than conventional TGA.

Additionally, we evaluate the thermal lag effect based on the termination temperature values of the three-step thermal decomposition (Figure 6, also Table 6). For cantilever-TGA, when the heating rate increases from 10 °C/min to 40 °C/min, the termination temperature values only change by 3.1 °C, 5.7 °C, and 12.6 °C for the three steps. In contrast, using conventional TGA, the termination temperature values change by 33.9 °C, 24.5 °C, and 76.4 °C, which are up to an order of magnitude larger. Again, these results verify that the thermal lag effect in the cantilever-TGA has been significantly minimized, thanks to the good thermal properties of silicon, miniature device size, and thermal isolation design.

## 6. Conclusions

In this work, we have demonstrated and analyzed the high-performance TG measurements using MEMS resonant cantilevers, with better accuracy and smaller thermal lags compared with conventional TGA. Through finite element modeling and experiments, we have verified the very fast thermal response of the resonant cantilevers of more than 10^4^ °C/s. Furthermore, we have conducted high-performance TG measurements of the thermal decomposition of Cu_2_(OH)_2_CO_3_ and CaC_2_O_4_·H_2_O. Compared with conventional TGA, our cantilever-TGA has exhibited better agreement with the theoretical weight loss values, with up to an order of magnitude smaller thermal lags at the same heating rate. The results suggest that the cantilever-TGA has better accuracy and smaller thermal lag, which holds promise for improving the TG characterization of materials in various fields.

## Figures and Tables

**Table 1:** Structural dimensions and material parameters in the finite element model.

Parameters	Value
Length of the cantilever (μm)	290
Width of the cantilever (μm)	140
Length of thermal isolation window (μm)	100
Width of thermal isolation window (μm)	100
Thickness of silicon layer (μm)	3
Thickness of molybdenum layer (μm)	0.1
Density of silicon (kg/m^3^)	2329
Density of molybdenum (kg/m^3^)	10,280
Thermal conductivity of silicon (W/(m·K))	150
Thermal conductivity of molybdenum (W/(m·K))	138
Heat Capacity of silicon (J/(kg·K))	700
Heat Capacity of molybdenum (J/(kg·K))	250
Boundary temperature (K)	293

**Table 2:** Heating power for reaching different temperature values.

Temperature (°C)	Heating Voltage (V)	Heater Resistance (Ω)	Heating Power (mW)
100	1.41	630.09	3.16
200	2.70	733.02	9.95
300	3.45	835.96	14.24
400	4.20	938.89	18.79
500	4.72	1041.83	21.38

**Table 3:** TG measurements of Cu_2_(OH)_2_CO_3_ at 5, and 20 °C/min. Total weight losses are compared with the theoretical value (Cantilever-TGA vs. Conventional TGA).

	Total Weight Loss wl_exp_ (%)		Deviation from Theoretical Value (wl_exp_ − wl_th_)/wl_th_	
	5 °C/min	20 °C/min	5 °C/min	20 °C/min
Cantilever-TGA	28.30	28.38	0.7%	1.0%
Conventional TGA	27.74	27.63	−1.3%	−1.7%

**Table 4:** TG measurements of Cu_2_(OH)_2_CO_3_ at 5, and 20 °C /min. Decomposition termination temperatures are compared (Cantilever-TGA vs. Conventional TGA).

	T (°C) (5 °C/min)	T′ (°C) (20 °C/min)	T′ − T (°C)
Cantilever- TGA	318.2	322.7	4.5
Conventional TGA	324.4	350.6	26.2

**Table 5:** TGA measurements of CaC_2_O_4_·H_2_O at 10, and 40 °C/min. Weight losses of three-stage decomposition are compared with theoretical values (Cantilever-TGA vs. Conventional TGA).

	Step#	Weight Loss wl_exp_ (%)		Deviation from Theoretical Value (wl_exp_ − wl_th_)/wl_th_	
		10 °C/min	40 °C/min	10 °C/min	40 °C/min
Cantilever-TGA	1	12.21	12.18	−0.9%	−1.1%
	2	19.14	19.57	−0.1%	2.1%
	3	29.21	28.63	−3.0%	−4.9%
Conventional TGA	1	11.74	11.34	−4.7%	−8.0%
	2	18.29	18.21	−4.5%	−5.0%
	3	30.67	31.10	1.9%	3.3%

**Table 6:** TGA measurements of CaC_2_O_4_·H_2_O at 10, and 40 °C/min. Decomposition termination temperatures at three stages are compared (Cantilever-TGA vs. Conventional TGA).

	Step#	T (°C) (10 °C/min)	T′ (°C) (40 °C/min)	T′ − T (°C)
Cantilever-TGA	1	164.2	167.3	3.1
	2	530.8	536.5	5.7
	3	751.6	764.2	12.6
Conventional TGA	1	173.5	207.4	33.9
	2	516.9	541.4	24.5
	3	743.7	820.1	76.4

## Data Availability

Not applicable.
